# Intratumoral characteristics of tumor and immune cells at baseline and on-treatment correlated with clinical responses to MPDL3280A, an engineered antibody against PD-L1

**DOI:** 10.1186/2051-1426-1-S1-O12

**Published:** 2013-11-07

**Authors:** Holbrook Kohrt, Marcin Kowanetz, Scott Gettinger, John Powderly, Hartmut Koeppen, Jeffrey A Sosman, Cristina Cruz, Yuanyuan Xiao, Ahmad Mokatrin, Gregg Fine, Daniel S Chen, F Stephen Hodi

**Affiliations:** 1 Stanford University Cancer Institute, Stanford, CA, USA; 2 Genentech Inc., South San Francisco, CA, USA; 3 Yale School of Medicine, New Haven, CT, USA; 4 Carolina BioOncology Institute, Huntersville, NC, USA; 5 Vanderbilt-Ingram Cancer Center, Nashville, TN, USA; 6 Vall d'Hebron University Hospital, Barcelona, Spain; 7 Dana-Farber Cancer Institute, Boston, MA, USA

## 

PD-L1, PD-L2 and other immune-related molecules regulate Th1 and Th2 immune responses. Tumor-expressed PD-L1, when bound to PD-1 or B7.1 on activated T cells, can mediate cancer immune evasion. While inhibiting PD-L1 receptor binding represents an attractive strategy to restore tumor-specific T-cell immunity, other immune-related factors and cell types within the tumor microenvironment affect anti-tumor T-cell responses. MPDL3280A, a human mAb containing an engineered Fc-domain designed to optimize efficacy and safety and to promote Th1-driven responses, is described here with PhI biomarker results. MPDL3280A was administered IV q3w in >300 pts with locally advanced or metastatic solid tumors. ORR was assessed by RECIST v1.1, including u/cCR and u/cPR. PD-L1 and CD8 were measured by IHC. The expression of ≈90 immune-related markers, including PD-L1 and PD-L2, were evaluated at baseline (BL) and on-treatment. BL tumor samples were available for 103 pts, and matched on-treatment samples were available for 26 pts. As of Feb 1, 2013, 140 pts enrolled prior to Aug 1, 2012 were evaluable for efficacy. Responses were observed in multiple tumor types including NSCLC (9/41), RCC (6/47), melanoma (11/38), CRC (1/4) and gastric cancer (1/1). An ORR of 21% (29/140) was observed in nonselected solid tumors with a duration of response of 1+ to 253+ days. Elevated pretreatment PD-L1 expression (IHC) was associated with response to MPDL3280A, and coordinated expression of PD-L1 and CD8+ T cells was seen. The ORR was 36% (13/36) for pts with PD-L1-pos tumors vs 13% (9/67) for pts with PD-L1-neg tumors. 81 pts had tumors evaluable for PD-L2. Median PD-L2 expression was ≈2x higher in PD-L1-pos tumors vs PD-L1-neg tumors. A T-cell gene signature (eg, CD8, EOMES, IFNγ, Granzyme A) was associated with treatment response. On treatment, responding tumors had increased PD-L1 expression and a Th1-dominant immune infiltrate, evidence of adaptive PD-L1 upregulation. Nonresponders showed minimal tumor CD8+ T-cell infiltration and an absence of T-cell activation. Additionally, elevated expression of immune suppressors, including FOXP3 and RORC, correlated with lack of response. Both the presence of Th1-related CD8 biology at BL and adaptive tumor PD-L1 enhancement with MPDL3280A treatment correlated with tumor response, as did PD-L1 status. Updated data will be presented.

